# 

**Published:** 1885-01

**Authors:** 


### INDEX

TO THE

### HOMŒOPATHIC PHYSICIAN.

### VOLUME V.

PAGE

Aconite........40, 70, 134, 229
Aconite. T. C. Hunter........ 322
" Lecture on. J. T. Kent, M. D. 69
American Institute Homœopathy, Discussion Present Status........ 46
Arnica diadema........ 386
Æthusia, Lecture on. J. T. Kent........ 246
Agaricus........ 40
All. cep........ 40
Ang........ 40
Agnus........ 40
Apis........40, 165
Arg-n........ 40
Am........40. 166, 199
Aloes, Remarks of Carroll Dunham on........ 352
Analysis, A Masterly........ 128
Arsen........12, 30, 199, 200
Arum. tri........ 164
Aurum........ 41
Baehr. Dr. Bernard........ 24
Berridge, E. W........ 149
Belladonna, Lecture on. Dr. J. T. Kent........ 177
Belladonna........36, 41, 134, 199, 229
Baryta carb........40, 129
Benz. ac........28, 40
Bryonia........200, 228
Cadmia........ 40
Caladium........40, 134
Calcarea phos........40, 200
Calc. ost........ 129
Camphora........ 40
Card........ 134
Cannabis sat........ 199
Cantharides........36, 130
Capsicum........ 40
Carbo. veg........12, 25, 139, 134
Caries of Teeth........ 225
Caruthers, R. E........ 109
Causticum........ 134
Central N. Y. Hom. Medical Society, Meeting of........166, 281
Chamomilla........ 169
Chilidonium........ 52
China........12, 134
Chronic Chills in Children. W. Steinrauf........ 359
Cholera, Are we to have, in U. S., in 1885? P. P. Wells........ 81
Cholera Microbes, etc. P. P. Wells........ 7
Cinchona........ 40
Cina in Spasmodic Cough. E. M. Hale. 115
Clinical Cases. E. W. Berridge........ 402
S. A. Kimball........ 258
R. B. Johnstone........ 147
E. Rushmore........ 149
Clinical Reflections. Ad. Lippe........ 232

PAGE

Clinical Duties. What they are and how they are to be Performed. P. P. Wells........ 333
Cocculus........ 40
Coffea........ 148
Colchicum........12, 40
Colocynth........ 200
Comparison of Diarrhœic Stools and accompaniment of Bryonia, etc. G. H. Clark........ 326
Comparison of Heart Symptoms Aggravated by Motion........ 256
Comparative Remarks........ 200
Conium........200, 201
Cuprum........12, 41, 200, 241
Condition of Stomach as Cause of Health and Disease. C. Pearson........ 286
Cyclopædia of Drug Pathogenesy. Ad. Lippe........ 211
Daphne........ 134
Digitalis........ 41
Diphtheria. J. V. Allen........ 153
Dose, Single or Second? R. R. Gregg........ 109
Dudgeon, Dr. R. E. Dr. Lippe's New Evidence for Curantur........ 61
Dunham, Carroll. Remarks on Aloes. 352
Equisetum........ 200
Eclecticism *vs.* Homœopathy. Dr. A. Lippe........ 271
Fifth Annual Session Hahn. Association, Announcement of........ 134
Foot-sweats. E. Fornias........ 129
Foote, G. F., Address before I. H. A..... 14
Ferrum........41, 147
Fluor. ac........ 41
Gregg, R. R., The Single or Second Dose........ 109
Hahnemann's Chronic Diseases. C. L. Swift........ 53
Hahnemann's Homœopathic Formula. Thos. Skinner........ 64
Honesty *vs.* Hypocrisy........ 75
Hahnemann's Three Precautions. C. W. Boyce........ 170
Hahnemann's Method of Individualization........ 252
Hamamelis........ 36
Helleborus........42, 130
Helonius........ 134
Hepar........ 42
Hints to Specialists. J. T. Kent........ 253
High Potencies and Dr. Jousset. B. Fincke, M. D........ 316
Homœopathic Medical Society of Pennsylvania........ 351
Ignatia........ 134
I. H. A. Notice........ 223

PAGE
Imagination of Disease as Indication in Prescribing. W. J. Guernsey....... 247
Involuntary Proving of Arnica. E. W. Berridge........ 386
Iodine........42, 129
Important Question Answered. H. C. Suess........ 101
Immaterial *vs.* Material. R. B. Johnstone........ 123
Johnstone, R. B. Clinical Case........ 78
J. T. Kent. Lecture on Aconite........ 69
Jatropha........ 12
Kali b........42, 164
Kali carb........42, 129, 134, 150
Kali iod........ 42
Kalmia........ 42
Lachesis, Proving by Induction of 7m, Fincke. B. Buchmann........ 394
Lecture on Cactus grandiflorus. J. T. Kent........ 314
Lecture on Cholera. C. G. Raue........ 235
" " Sulphur. J. T. Kent........ 140
" " Abrotanum........ 76
Lachesis, Physiological Proving of. B. Fincke........ 90
Lippe, Ad.
The Cholera........ 10
Law of Similars........ 356
Lummis, M. D........ 107
Lippe's New Evidence for "Curantur." R. E. Dudgeon........ 61
Masterly Analysis, A........ 128
Millspaugh, American Medicinal Plants. B. & T........ 260
Moses' Regulations as to Organs of Generation........ 393
Magnesia-carb........42, 134
Mercurius........ 42
Medor rhinum........ 149
Mercurius-iod........ 165
Merc. cyan........ 164
Murex........ 182
Merc. Bin.-iod........ 165
Morphine........ 166
Nephralgia. E. J. L........ 199
New Repertory........ 163
Nut for Dr. Dudgeon to Crack........ 116
Natrum sulph........ 42
Nicotin........ 13
Nitric acid........42, 129, 165
Nux vomica........168, 182, 200
Ocular Surgery........ 285
Obituaries........ 282
Obituary........ 325
Ocimum........ 200
Organon in Homœopathic Colleges........ 13
Ol. animale........ 134
Pareira........42, 200
Phos........ 42
Phos. acid........43, 134, 168
Phytolacca........43, 121, 164
Plumbum........130, 200
Podophyllum........130, 150, 182
Psorinum........25, 43
Pulsatilla........43, 182
Psora and Syphilis. Dr. Wolf........ 96
Peculiar Eye Symptoms........ 134
Pursuit of Knowledge under Difficulties. P. P. Wells........ 117
Proceedings of Central New York Homœopathic Society........166, 281
Proving of Convallaria Majalis. I. J. Lane........ 292
Polygonum Hydropiper........ 328
Pneumonia........ 361

PAGE
Progressive Medicine, Review of........ 259
Proving of Apiol. S. Swan........ 345
Pulsatilla, Lecture on. J. T. Kent........ 215
Physician's Duty........ 37
Query, A. O. W. Smith........ 139
Remarks on Aloes. C. Dunham........ 352
Remedies as Timekeepers. C. C. Smith........ 385
Repetition of Dose in Treatment........ 184
Reflections........ 389
Rushmore, Edward. Surgery or Medicine?........ 38
Ranunculus B........ 134
Rhus tox........43, 168
Ruta........ 43
Sanguinaria nit. J. S. Smith........ 385
Sepia, Some Uses of. W. E. Leonard... 245
Some Urinary Troubles. B. Ehrman.. 382
Surgery or Medicine? E. Rushmore... 38
Sabadilla........ 43
Sabina........ 43
Sambucus........ 43
Sarsaparilla........ 43
Seneg........ 134
Sepia........129, 175, 182
Silicia........39, 43, 129, 134, 166
Spigelia........ 166
Staphisagria........43, 200
Strammoniums........ 200
Strontinum........ 43
Squilla........ 130
Sulphur........12, 25, 39, 43, 129, 168, 360
Sulphuric acid........ 200
Tabac........ 200
Tannic acid........ 42
Theridium........ 43
Therapeutics of Bone Diseases. L. B. Wells........ 40
Therapeutical Hints. C. Hering........ 112
The Organon in our Colleges. M. D. Lummis........ 107
Therapeutics of Angina........ 396
Three Cases of Purpura Hæmorrhagica. G. W. Winterburn........ 306
Thirst Symptoms........ 250
Thrombidium........ 233
Thuja........43, 129, 134
Triosteum........ 43
Triolidium........ 149
Unkind Criticism........ 257
Undeveloped Case. J. T. Kent........ 25
Urinary Troubles, Some. B. Ehrman 382
Veratrum viride........ 12
Vinca........ 43
Viola odorata........ 129
Wells, P. P.
Status of American Institute........ 46
Cholera, Are We to Have it?........ 81
Pursuit of Knowledge Under Difficulties........ 117
In Whose Eye is the Mote?........ 131
Differentiation of Remedies........ 189, 225, 297
Medical Education and Acceptance of Homœopathic Law........ 369
What is Homœopathy? J. T. Kent..... 346
Warts Cured. E. W. Berridge........ 113
What I Know of Phytolacca. E. B. Nash........ 121
Who is Our Guide, and Where Shall We Find Him? Dr. Dunn........ 74
Wolf. Psora and Syphilis........ 90
Women's Homœopathic Association of Pennsylvania........ 210
Zinc........134, 139

